# Exploring the Feasibility of Paper-Based Substrates for User-Friendly Electrochemiluminescent Sensors

**DOI:** 10.1021/acs.analchem.5c06606

**Published:** 2026-01-30

**Authors:** Panagiota M. Kalligosfyri, Luca Scognamiglio, Elena Sossich, Ningtao Cheng, Federico Polo, Stefano Cinti

**Affiliations:** † Department of Pharmacy, University of Naples “Federico II”, Naples 80131, Italy; ‡ Department of Molecular Sciences and Nanosystems, 19047 Ca’ Foscari University of Venice, Via Torino 155, Venice 30172, Italy; § School of Public Health, 26441 Zhejiang University School of Medicine, Hangzhou, Zhejiang 310058, China; ∥ European Centre for Living Technology (ECLT), Ca’ Bottacin, Venice 30124, Italy; ⊥ Sbarro Institute for Cancer Research and Molecular Medicine, Center for Biotechnology, College of Science and Technology, Temple University, Philadelphia, Pennsylvania 19122, United States; # 9307 Bioelectronics Task Force at University of Naples Federico II, Via Cinthia 21, Naples 80126, Italy

## Abstract

Electrochemiluminescence (ECL) is a light-emitting process in which electrochemically generated excited states relax to the ground state and emit photons; in many systems, this excitation is produced through reactions involving a coreactant. ECL has been successfully employed in devising analytical methodologies requiring exceptional sensitivity and ultralow background, critical for precise measurements. Its seamless integration with paper-based platforms further enhances easiness of use, portability and cost-efficiency, expanding its applicability across biomedical, environmental, and point-of-care fields. This novel study aims at integrating the sensitivity offered by ECL and the experimental convenience of paper-based substrates, thus bridging a gap in the science of measurements and providing a new analytical tool with relevant features. Office paper and filter paper were evaluated and compared to conventional and widely used polyester-based screen-printed electrodes. The main goal was to assess their electrochemical behavior, analytical performance and reliability for long-term reagent storage. In fact, the use of paper might lead to a key innovation in the field: tripropylamine, the sacrificial coreactant successfully employed in combination with the luminophore tris­(2,2′-bipiridine)­ruthenium­(II), namely Ru­(bpy)_3_^2+^, in a model ECL system, was dried by taking advantage of the paper’s porosity. This allowed eliminating manual reagent addition/mixing during analysis, with the result of simplifying the experimental procedure at the end-user and enhancing storage stability. As promising results, the electrochemical characterization revealed that office paper exhibited superior sensor’s performance due to its lower porosity, while the highly porous filter paper caused signal loss and reduced analytical performance. Features such as sensitivity, stability, repeatability and storage ability were assessed. Overall, this study demonstrates the potential of paper-based substrates, especially commercial office paper, as sustainable and cost-effective platforms for ECL sensing.

## Graphical abstract

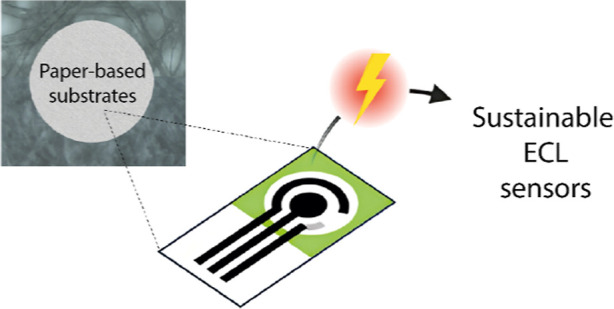

## Introduction

Electrochemiluminescence (ECL) is a light-emitting process in which electrochemically generated species react near the electrode surface to produce excited-state luminophores that relax to the ground state and emit photons; this typically occurs in solution by applying a specific potential, often in the presence of a sacrificial coreactant.^1^ ECL has been successfully employed in devising analytical methodologies and tools requiring exceptional sensitivity, ultralow background, and broad dynamic ranges spanning up to 6 orders of magnitude.^2–4^ Current ECL platforms, even employing new concepts such as nanocomposites for ECL emission amplification,^5^ typically rely on rigid electrodes, such as platinum, gold, glassy carbon or silicon, integrated into automated analyzers or well-plates. However, while their efficacy for detecting biomarkers like hormones, proteins, toxins and coagulation indicators^2,4^ is widely demonstrated, the cost, complexity and low-portability might represent a limitation in resource-constrained settings.^6–8^ The reliance on nondisposable, rigid components contrast with the growing demand for eco-friendly analytical platforms, highlighting the need for more sustainable alternatives in sensor design, and the requirement for multielectrode setups further complicate the whole context, increasing material consumption and electronic waste.^4,9,10^

The integration of paper-based substrates with ECL technology might offer a transformative alternative to conventional electrode materials.^11–14^ Paper’s inherent properties, biodegradability, capillary-driven fluidics, and ultralow cost, align with the need for sustainable point-of-care (POC) single-use analytical devices.^12–15^ ECL has been coupled to various systems, for instance lateral flow assays, as recently reported for the detection of *Mycobacterium tuberculosis* and SARS-CoV-2.^16,17^ More specifically, paper-based ECL immunoassays have been developed by integrating lateral flow configurations composed of absorbent pads and nitrocellulose membranes, where specific antibodies were immobilized and combined with commercial screen-printed electrodes.^16,17^ After target detection, the readout was performed via ECL measurements to enhance the sensitivity of lateral flow assays, which are typically limited to qualitative results. In both approaches, however, the ECL architecture was not inherently related to the paper-based assay. In another work, an origami-type filter paper (Whatman No. 2) sensing interfaces have been employed for environmental monitoring and microbial assays, using ECL as the quantitative method: herein, the origami paper-based substrate was folded, with each layer containing reagents, and detection of lead ions occurred at a homemade SPE placed onto last layer.^18^ Following a similar low-cost approach for early cancer diagnosis, an integrated paper-based bipolar electrode ECL device used patterned hydrophobic and hydrophilic zones to perform target detection via immobilized DNA probes. The recognition elements were immobilized in defined zones, and a self-cleaning system channels buffer to rinse the pencil-drawn electrodes. This specific configuration ensured excess fluid absorption, prevented contamination and achieved reproducible measurements.^14^ Additionally, efforts have been made to perform an ECL assay within the paper layer as reported by Feng et al. while detecting pathogenic bacteria food samples. This example was characterized by the necessity of cutting the paper-based area before transferring it onto an external indium tin oxide electrode.^19^ This approach, however, limits the simplicity and the concept of an all-in-one platform.

Taking into consideration most of the reported ECL studies employing paper-based substrate, it is clear how the latter ones offer customizable and low-cost possibilities for biosensing applications. However, they are often limited to proof-of-concept studies using noncustomizable materials, frequently require external electrodes, and lack systematic characterization of their properties such as porosity, fiber structure, and surface chemistry.^16–20^ These factors can result in uncontrolled fluid spreading, heterogeneous electrochemical responses and reduced reproducibility. In fact, slight attention has been paid to evaluating different paper types as substrates for both electrochemical detection and reagent storage. To address this gap, our study presents the first direct comparison of two widely available, low-cost papers, namely filter paper and office paper, within an integrated device architecture (different to the use of ECL as an external readout system only). Taking advantage of the paper’s porosity, screen-printed ECL sensors were fabricated directly on these substrates by immobilizing via a simple drop-casting tripropylamine (TPA), the sacrificial coreactant successfully employed in combination with the luminophore tris­(2,2′-bipiridine)­ruthenium­(II), namely Ru­(bpy)_3_^2+^, in a model ECL system.^1,21^ This approach enabled the fabrication of ready-to-use sensors that require no additional reagents handling or preparation, simplifying the analytical workflow for nonspecialized users and in potential decentralized contexts. The electrochemical characterization carried out in this study has revealed that the porosity and morphology of the paper-based substrate strongly influence performance, while calibration experiments demonstrated linear ECL responses for all papers interrogated, thus confirming their suitability for quantitative analysis. Office paper was further selected as an all-in-one platform substrate because of its superior experimental performance. Storage stability tests showed that TPA-functionalized electrodes retained robust ECL activity after 10 days under ambient conditions, highlighting their potential as disposable, accessible, and reliable devices for POC and field-based applications.

The novelty of our approach lies not only in demonstrating that paper can be used for ECL-based sensing, but also in identifying which type of paper is more suitable under specific conditions. By providing this comparative assessment, we address a critical gap in the literature: the lack of systematic evaluation of paper-based substrates in developing ECL devices. Overall, this work highlights office paper as a promising, sustainable substrate for ECL sensor development and demonstrates the benefits of reagent immobilization in enhancing usability and reliability. These findings pave the way for the rational design of portable, low-cost paper-based ECL sensing platforms with broad applicability in clinical diagnostics, environmental monitoring, and food safety.

## Experimental Section

### Materials and Methods

Tris­(2,2′-bipyridine)­ruthenium­(II) chloride (Ru­(bpy)_3_^2+^), tri-*n*-propylamine (TPA) and all the common reagents were purchased by Sigma-Aldrich (St. Louis, MO, USA). Office paper obtained from local store was used as a paper-substrate for screen printing and reagent storage. Whatman grade 1 filter paper (Merck KGaA, Darmstadt, Germany) with a wicking speed of 150 s/100 mL, a thickness of 180 μm, and a pore size of 11 μm. The bottom part of the paper-substrate was laminated with the use of an adhesive tape. The upper part of the paper-substrates was pretreated by wax printing for the creation of hydrophilic areas using the ColorQube 8580 office printer obtained from Xerox (USA).

### Screen-Printed Electrode Preparation

The screen-printed electrodes (SPEs) were fabricated in-house, as previously reported.^22,23^ The flexibility of the electrodes was attributed to the use of a flexible polyester substrate as the printing platform. Conductive silver/silver chloride ink was used for the reference electrode while carbon ink was used for the printing of the working and counter electrodes. The SPEs were treated at 100 °C for 30 min to dry and stabilize the ink. Prior to each measurement, adhesive tape was applied to the electrochemical sensor to prevent spreading and leakage of the sample, ensuring proper functioning of the electrical components and creating a hydrophobic boundary that defined the sample deposition area. For the paper-based electrodes the previously reported procedure was followed.^24,25^ Briefly, a pretreatment step using wax-based ink is required to create hydrophobic barriers on the substrate. This step is essential to prevent sample spreading and to facilitate proper sample handling. The process started with designing the desired pattern in Adobe Illustrator, which was then printed onto the paper using a wax printer. The printed wax was cured at 100 °C for 30 s, allowing it to melt and penetrate the paper matrix, forming stable hydrophobic barriers that control fluid flow within the sensing area. Following this, the conductive inks were printed, and the remaining fabrication steps proceeded similarly to those used for polyester-based SPEs.

### Cyclic Voltammetry and ECL Measurements

Electrochemical and ECL measurements were performed using a μStat ECL potentiostat (Metrohm DropSens Italia Srl, Origgio, Italy) and the results were visualized via a portable computer with the dedicated application DropView (8400). To characterize the polyester- and paper-based SPE the cyclic voltammetry (CV) experiments were carried out by employing 5 mM K_3_[Fe­(CN)_6_] as the redox mediator in deionized water with 100 mM KCl as the supporting electrolyte. Concerning the ECL experiments, 40 μL of sample, containing the desired concentration of Ru­(bpy)_3_^2+^ and a final concentration of 10 mM TPA, was used for each measurement. ECL served as the primary analytical detection method throughout the project. ECL was efficiently detected and analyzed by means of photodetectors integrated within the electrochemical cell (Metrohm DropSens) and connected to the external channel of the potentiostat. The measurements were performed with a potential step of 1 mV and a scan rate of 0.05 V/s, over a potential range of +0.8–+1.8 V (vs the screen-printed Ag/AgCl reference electrode), with a single scan per measurement. Signal amplification was set at ×100.^26,27^ These conditions were chosen to maximize the analytical performance and consistency of the ECL signals obtained.

### TPA Immobilization and ECL Measurements

In the proposed paper-based ECL system, Ru­(bpy)_3_^2+^ acts as the luminophore while TPA is immobilized near the electrode. Upon applying a suitable potential range, both Ru­(bpy)_3_^2+^ and TPA are oxidized at the electrode to form Ru­(bpy)_3_^3+^ and the TPA^•^ radical, respectively. The TPA^•^ radical then reduces Ru­(bpy)_3_^3+^ to the excited state Ru­(bpy)_3_^2+^*. As the excited luminophore relaxes back to its ground state, it emits orange light at approximately 620 nm (Figure S1, Supporting Information).^28^ To simplify the analytical workflow and enhance end-user accessibility, an immobilization strategy was employed to preload TPA onto the working electrode surface (Figure 1).

**Figure 1:**
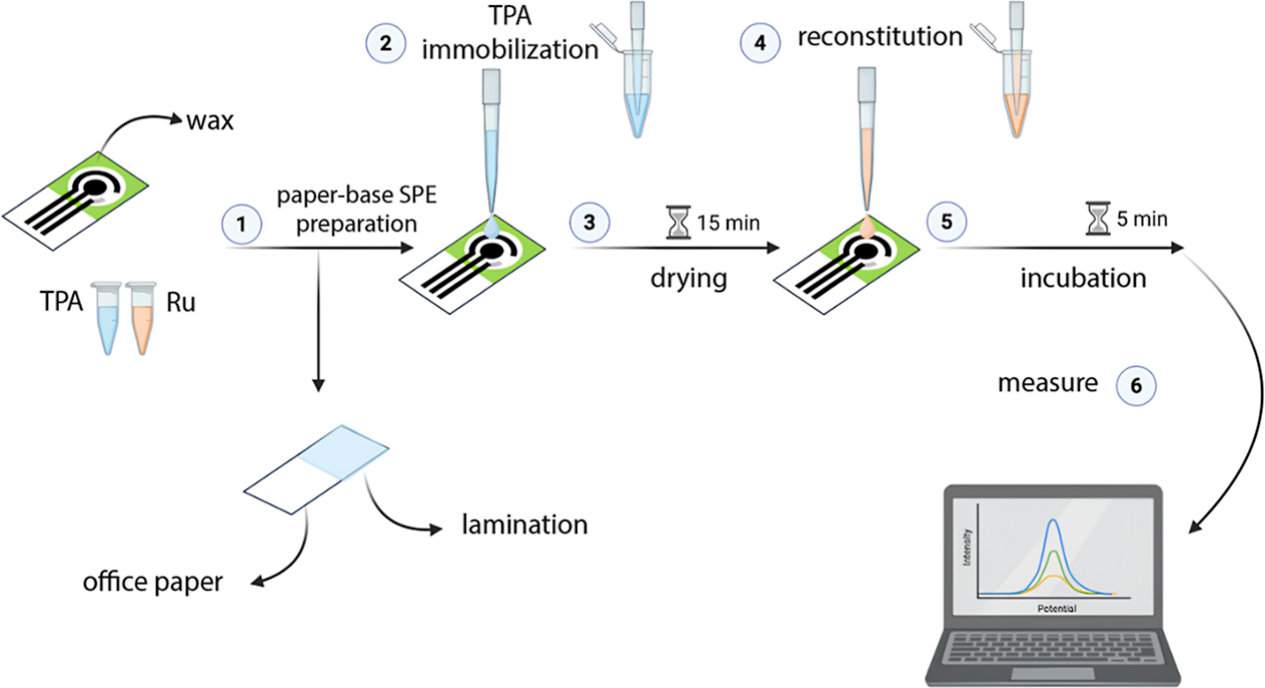
Workflow of the preparation of the ready-to-use paper-based SPEs. Initially the prepared paper-based SPE was laminated. A 2 M TPA solution (final immobilized concentration 200 mM) was drop-cast onto the working electrode and dried at room temperature for 15 min. For measurements, 50 μL of Ru­(bpy)_3_^2+^ solution was added and incubated for 5 min before performing ECL analysis.

This approach significantly reduces the number of operational steps during analysis, eliminating the need for manual reagent handling. Due to the inherent porosity of the screen-printed paper electrodes, uncontrolled spreading of the solution was a potential issue. To address this, the reverse side of each electrode was carefully laminated with adhesive tape. This lamination step was essential for confining reagent deposition strictly to the working electrode area, thereby ensuring accurate and reproducible assay conditions. Following optimization, 5 μL of a 2 M TPA solution (resulting in a final immobilized concentration of 200 mM) was precisely drop-cast onto the working electrode surface. This specific volume and concentration were chosen to achieve optimal analytical performance while maintaining the structural integrity of the electrode. After deposition, the electrodes were thoroughly dried at room temperature in a fume hood for approximately 15 min. This drying step was crucial to ensure uniform reagent distribution and stable immobilization. For measurement, 50 μL of a Ru­(bpy)_3_^2+^ solution was added to the electrode and incubated for 5 min. The ECL measurement procedure was then carried out as previously described.

## Results and Discussion

### Morphological Characterization of the Paper-Based Substrates

To investigate the porosity and morphology of the paper-based substrates, microscopic analysis was carried out using an Exacta Optech microscope equipped with a Euromex Scientific Camera (Exacta Labcenter SpA, Italy). Images were obtained after heat pretreatment of both filter paper and office paper. The two papers, namely office and filter paper, were used as potential substrates for the fabrication of the screen-printed electrochemiluminescent sensors, owing to their ease of use, accessibility and low cost. Both paper substrates were wax-treated to create well-defined hydrophobic barriers, which provide liquid control preventing sample loss. Furthermore, each substrate was laminated on the back side using adhesive tape to provide mechanical support and improve durability during handling and measurements. The filter paper exhibited a more open and porous structure, characterized by larger voids between fibers (Figure 2A,B), which can facilitate fluid penetration and enhance mass transport. In contrast, the office paper showed a denser fiber network with fewer visible pores, suggesting higher mechanical integrity and smoother surfaces (Figure 2C,D). These structural differences are consistent with the electrochemical characterization, which confirmed that office paper offers a favorable balance of conductivity and structure, while the excessive porosity of filter paper limited its electrochemical performance. Thus, while filter paper provides enhanced analyte diffusion, office paper appears to be better suited for stable and reproducible sensing applications.

**Figure 2:**
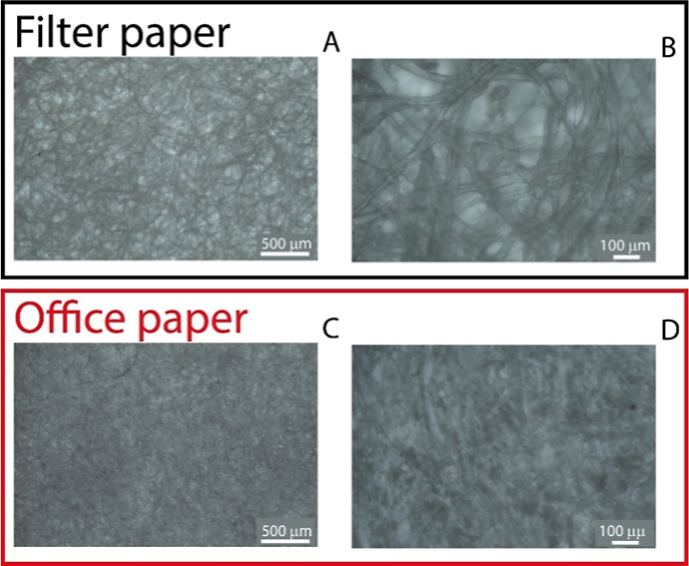
Microscopic analysis of the paper-based substrates at two magnifications. Filter paper: (A) 4×, (B) 20× magnification. Office paper: (C) 4×, (D) 20× magnification.

### Electrochemical Characterization of the Screen-Printing Substrates

The primary objective was the electrochemical characterization of the substrates used for the screen-printing of the electrochemical sensors. Polyester- and the two paper-based substrates, namely standard office paper and Whatman filter paper grade 1, were tested and compared. Polyester was also characterized as a comparison with the paper-based substrates because of its flexibility, robustness and wide usage.^22,29,30^ The electrochemical characterization was carried out by CV and the results for polyester, office and filter-based systems are shown in Figures S2–S4 of the Supporting Information, respectively. The characterization process was carried out with 5 mM K_3_[Fe­(CN)_6_] as the redox mediator in deionized water with 100 mM KCl as the supporting electrolyte. Initially, the measurements were performed by varying the scan rates in the range 0.02–0.5 V/s. CVs show that the peak current intensity for both the anodic and cathodic processes increases linearly with the square root of the scan rate, witnessing that the heterogeneous electron transfer process to the redox mediator is under diffusion controlled kinetics. The large peak width and peak-to-peak separation, commonly observed with this redox mediator, is mainly due to the slow electron transfer kinetics and the ohmic drop, which cannot be successfully suppressed with the current instrument. However, the results demonstrate the successful fabrication of the electrodes.

### Optimization Studies

Following the electrochemical characterization, optimization studies for the Ru­(bpy)_3_^2+^/TPA system were performed. In particular, we investigated the dependence of the ECL output on the incubation time and the TPA concentration. The reaction time leading to ECL was the first parameter to be optimized. To this end, a solution containing 0.1 mM of Ru­(bpy)_3_^2+^ and 50 mM of TPA was employed and tested on polyester substrate. The incubation time was evaluated over a range of 1–15 min. As shown in Figure 3, the ECL signal generated across the different incubation times was comparable; however, the 5 min time interval yielded the most reproducible results, as witnessed by the small standard deviation of the triplicate measurement. Therefore, 5 min were selected as the optimal incubation time for the reaction to occur.

**Figure 3:**
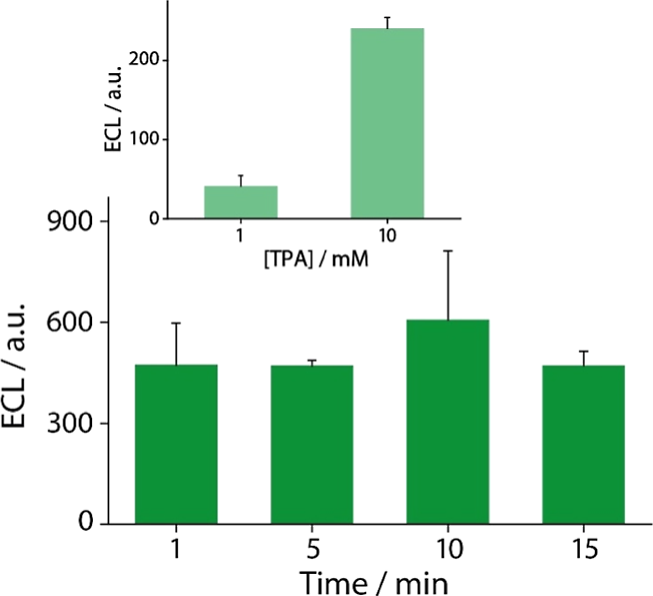
Optimization of the incubation time in solution. The histograms represent the sensor’s signal response at various incubation time intervals. Solutions of 0.1 mM of Ru­(bpy)_3_^2+^ and 50 mM of TPA were employed for this study. The inset is showing the optimization of the TPA concentration. The histograms represent the sensor’s signal response at 1 and 10 mM of TPA. Solution of 0.25 mM of Ru­(bpy)_3_^2+^ was employed for this study. The experiments were performed in triplicates.

The second optimized parameter concerns the concentration of TPA. To this aim, a solution containing 0.25 mM Ru­(bpy)_3_^2+^ was prepared, and four different concentrations of TPA, namely 1, 10, 50, and 100 mM were tested. Although higher TPA concentrations (specifically 50 mM and 100 mM) could lead to stronger signals, the measurements at these levels were not reproducible. Therefore, they are not shown in Figure 3. We observed that the increased TPA concentrations altered the viscosity of the solution. The higher viscosity caused the sample to spread unevenly within the designated chamber of the ECL instrument, which in turn led to inconsistent measurements. When considering the overall low volume of the electrochemical cell provided by the polyester- and paper-based SPE, the above-mentioned issue can indeed affect the reproducibility of ECL activation mechanism. Therefore, only the 1 mM and 10 mM TPA concentrations were considered for a detailed comparison of the sensor’s performance (inset of Figure 3). Among these two, 10 mM TPA was found to be the optimal concentration, offering both a stronger signal and reliable reproducibility.

### Analytical Performance

Following the optimization studies, the analytical performance of the newly developed method was thoroughly evaluated for the polyester- and the two paper-based substrates to identify the most suitable option to fabricate the integrated storage and detection platform. This was achieved by measuring the ECL response using a series of increasing concentrations of Ru­(bpy)_3_^2+^. To ensure reliability and reproducibility, all measurements were conducted in triplicates. Each experiment followed an incubation time of 5 min and was carried out using all the optimized conditions previously described. The analytical characteristics of each substrate, including their ability to support consistent signal generation and maintain structural integrity during the measurement process, were considered. This comparison was critical for selecting the optimal substrate for the fabrication of a reliable and efficient all-in-one ECL-based sensor system.

#### Polyester Substrate

The polyester substrate was used as the reference material for comparison with the paper-based substrates. Electrochemical sensors were fabricated on the polyester substrate following the screen-printing procedure described in detail in the experimental section. To evaluate the analytical performance of the system, a set of calibrator solutions containing Ru­(bpy)_3_^2+^ in the range of concentration 0.25–5 μM, along with a constant concentration of 10 mM TPA, were prepared and tested. The measurements revealed a dynamic range up to 5 μM and a linear response within the concentration range 0.25–2.5 μM, thus indicating the optimal operating range of concentration in which the sensor provides reliable and proportional signal output (Figure S5).

The resulting calibration curve, *y* = 356.57*x* – 8.03, represents the measured change in signal intensity (*y*) against the concentration of Ru­(bpy)_3_^2+^ (*x*). It provides a coefficient of determination (*R* ^2^) of 0.98, demonstrating a strong linear correlation between signal intensity and analyte concentration.

#### Paper-Based Substrates

To adapt the two paper-based substrates for electrochemical sensing applications, an additional processing step was necessary, as thoroughly described previously.^31^ Briefly, hydrophobic barriers were introduced to prevent sample spreading and ensure controlled liquid confinement on the electrode surface. These barriers were created by wax-printing: patterns were designed in Adobe Illustrator, printed using a solid-ink printer (ColorQube 8580, Xerox, USA), and thermally cured at 100 °C for 2 min to melt the wax and form effective hydrophobic boundaries.

The analytical performance of both paper-based substrates was evaluated and compared. This included testing the linearity of the ECL response using the optimized experimental conditions and solution preparations described in the previous section. The aim was to determine whether the paper-based SPEs could produce a consistent and proportional signal response to varying concentrations of Ru­(bpy)_3_^2+^ in the presence of 10 mM TPA. The results showed that both office paper (Figure 4A) and filter paper (Figure 4B) exhibited a clear linear relationship between the Ru­(bpy)_3_^2+^ concentration and the resulting ECL signal intensity, with the ECL signal increasing as the Ru­(bpy)_3_^2+^ concentration increased (Figure S6).

**Figure 4:**
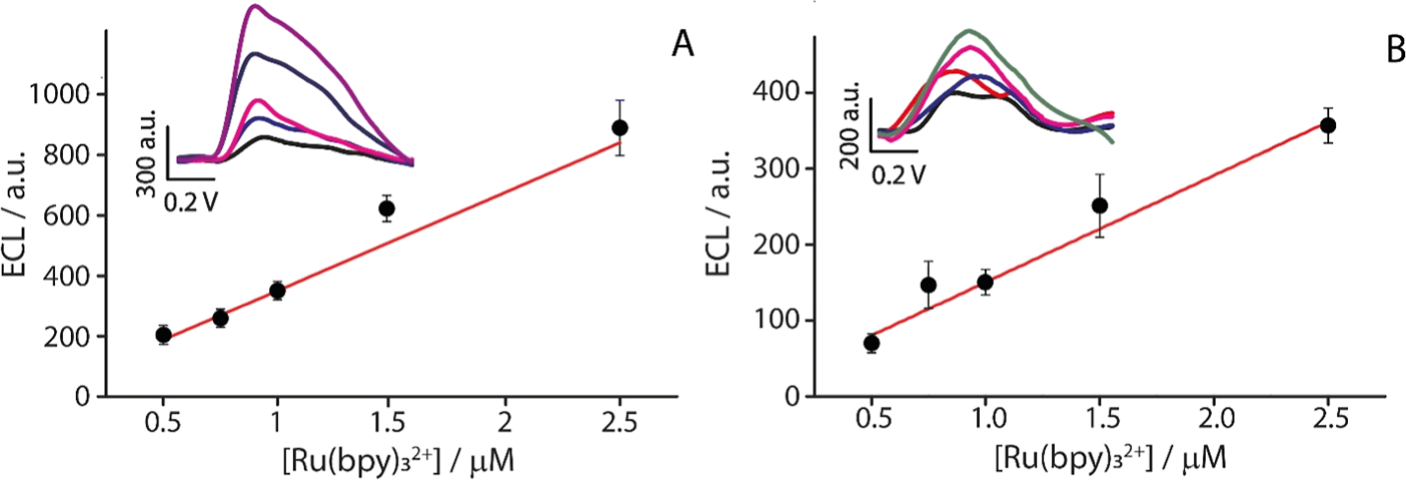
(A) Calibration curve obtained with the office paper-based SPEs at increasing concentrations of Ru­(bpy)_3_^2+^ in the range 0.5–2.5 μM. Inset (Figure S6): ECL signal obtained from the increasing concentrations of Ru­(bpy)_3_^2+^. (B) Calibration curve obtained by the filter paper-based SPEs at increasing concentrations of Ru­(bpy)_3_^2+^ in the range 0.5–2.5 μM. Inset: ECL signal obtained from the increasing concentrations of Ru­(bpy)_3_^2+^. The ECL measurements were performed in solution, in the presence of 10 mM of TPA. The experiments were performed in triplicates.

This indicates that the analytical system maintained a linear behavior when implemented on paper-based platforms, which is essential for reliable quantitative analysis. The corresponding linear equations describing the system’s response for each substrate are as follows: *y* = 326.94*x* + 12,51 with *R* ^2^ = 0.97 for office paper; *y* = 103,04*x* + 67,29 with an *R* ^2^ = 0.98 for filter paper. As shown in Figure 4, office paper demonstrated higher sensitivity when used as a substrate for SPEs, as witnessed by a higher slope value. This linear response confirms the sensor’s ability to quantitatively detect Ru­(bpy)_3_^2+^ within the investigated concentration range under optimized conditions.

While office paper provided a favorable balance of conductivity and structure, filter paper’s excessive porosity^13^ likely contributed to analyte absorption and reduced accessibility of the working surface, ultimately limiting its performance. Specifically, regarding the spatial distribution of ECL, the results indicate that paper’s porosity strongly affects the emission profile. Because in the proposed ECL system the detector captures light from above the drop, the measured signal represents the integrated emission from this layer across the entire drop area rather than from a single point. This means that the higher porous substrate, i.e. filter paper, allow the luminophore and coreactant to diffuse deeper into the paper matrix, reducing the concentration near the electrode and thus decreasing the detected ECL intensity. In contrast, the less porous substrates, i.e. office paper confine the reactive species closer to the electrode, producing stronger, more uniform emission detectable by the photodiode. Therefore, office paper displayed sensitivity comparable to polyester substrates while offering more reproducible ECL responses (Figure S7). This result underscores its potential as a low-cost and functional alternative to conventional materials, supporting its selection for further investigations.

### Optimization and Analytical Characterization of the Ready-to-Use Paper-Based SPEs

Based on the results presented above, we selected office paper as the substrate to further explore its applicability as a ready-to-use sustainable ECL platform. Its combination of high sensitivity, reproducibility, and readily available nature makes it a practical choice for widespread applications. This accessibility ensures that the developed sensors can be easily adopted in diverse settings. The present study was carried out following the procedure outlined in the Methods Section. A stock of 98% liquid TPA was used, and all working solutions were prepared immediately prior to use. The 5 M TPA concentration was used without further dilution, while all the other concentrations were prepared via serial dilution to ensure consistent and reproducible results. This rationale guided the selection of TPA concentrations for immobilization. For this study, increasing concentrations of TPA were immobilized, specifically 0.5, 1, 2, and 5 M, which were subsequently rehydrated with 50 μL of a Ru­(bpy)_3_^2+^ solution at a concentration of 3.5 μM. This approach resulted in final TPA concentrations on the working electrode (WE) surface of 50, 100, 200, and 500 mM, respectively.

In parallel, the TPA solution was also tested for immobilization on polyester-based electrodes for comparison. Across the tested concentration range of 10–200 mM, the resulting ECL signal intensity varied between 110 and 40, which is considered relatively low for the given Ru­(bpy)_3_^2+^ concentration. The reduced performance is primarily attributed to the highly hydrophobic nature of the polyester substrate, which further highlights the paper’s porosity as the optimal feature for reagent storage and the preparation of ready-to-use platforms. As the TPA concentration increased, it became more difficult to immobilize the solution effectively on the electrode surface due to excessive spreading, which compromised signal generation and reproducibility.

Similarly, for the office paper substrate, the use of 5 M TPA (equivalent to 500 mM on the WE) was excluded from further analysis. This concentration yielded a low signal and poor reproducibility, likely due to spreading effects and high coffee ring drying effects or unfavorable interactions with the substrate surface. Our findings, shown in Figure 5A, indicate that the optimal drop-casted TPA concentration on the office paper substrate is 2 M, which corresponds to 200 mM after reconstitution and provides the best response.

**Figure 5:**
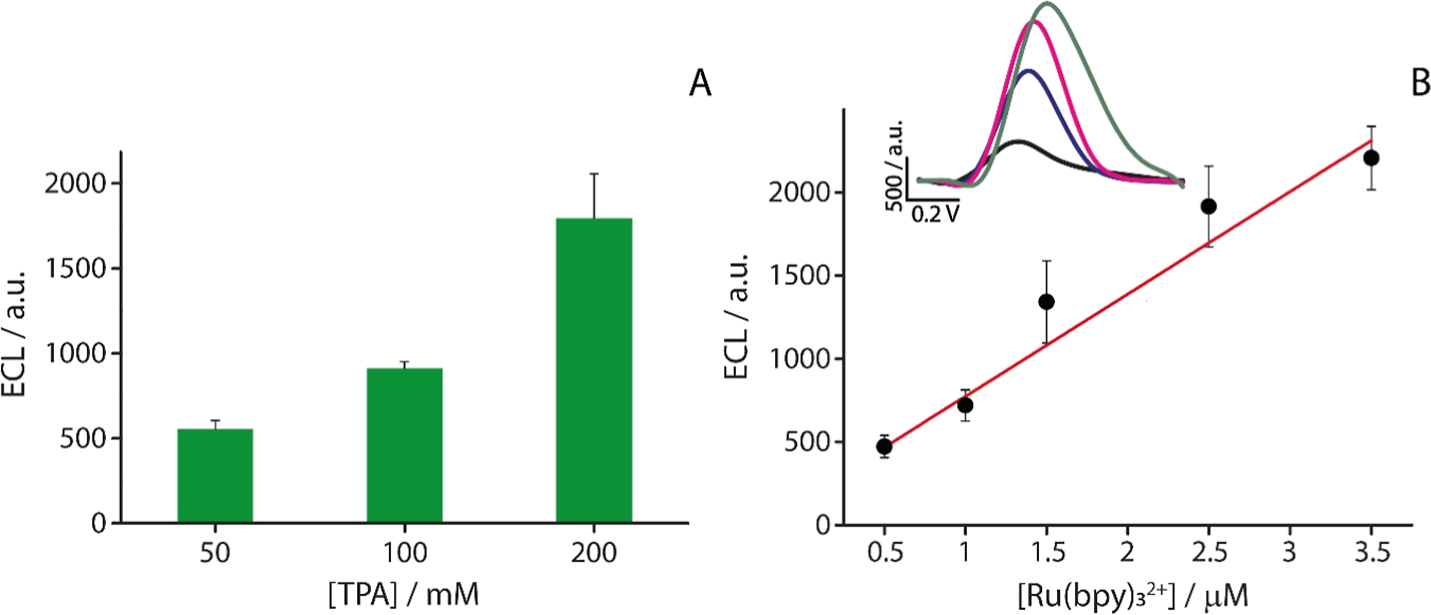
(A) Optimization of the concentration of TPA immobilized on the WE on the office paper substrate. The immobilized TPA was tested in the presence of 3.5 μM Ru­(bpy)_3_^2+^. (B) Calibration curve obtained for the office paper-based SPEs, with 2 M immobilized TPA, at increasing concentrations of Ru­(bpy)_3_^2+^ in the range of 0.1–1 μM. Inset (Figure S8): ECL signal obtained from the increasing concentrations of Ru­(bpy)_3_^2+^. The experiments were performed in triplicates.

To conclude the optimization studies, office paper was further characterized to evaluate the sensing performance of the ready-to-use ECL platforms. Specifically, calibration curve was generated for the office substrate. The goal was to assess the linearity of the electrochemical response and determine the sensitivity of the platforms in response to increasing concentrations of the Ru­(bpy)_3_^2+^ in the range of 0.5–3.5 μM. The ECL peak appears at approximately 1.1–1.2 V (Figure S8), with slight shifts observed at higher Ru­(bpy)_3_^2+^ concentrations. This behavior is partly due to diffusion effects and the use of a pseudoreference electrode (screen-printed Ag/AgCl reference electrode), which can introduce small potential variations.^32^ The calibration curve obtained using office paper as the substrate is shown in Figure 5B and features the equation *y* = 614.71*x* + 159.81 with an *R* ^2^ = 0.96. Remarkably, this system offers a LOD as low as 0.39 μM.

These results indicate that the paper-based system successfully produced linear responses to increasing analyte concentrations, confirming the functionality of the immobilized TPA in triggering the ECL reaction. Overall, office paper exhibited effective ECL behavior, reinforcing the potential of paper-based materials for sensor development. Consequently, we proceeded to evaluate the storage stability office paper-based SPEs.

### Storage Stability of the Ready-to-Use SPEs

To further assess the practical feasibility of this approach, stability tests were conducted to evaluate the performance of TPA-immobilized electrodes after prolonged storage. Office paper-based electrodes, selected for their promising analytical performance and measurement reproducibility, were prepared as follows: (1) once the paper substrate was laminated on the rear side, as described in the Experimental Section, a 2 M solution of TPA was carefully drop-casted onto the working electrode area. This corresponds to a final concentration of 200 mM of TPA once reconstituted. (2) The electrodes containing the immobilized TPA were left to dry under ambient conditions in a fume hood at room temperature. This ensured gradual evaporation of the solvent without compromising the integrity of the substrate or the deposited material. (3) Once fully dried, the paper-based SPEs were stored in resealable (zip-closed) plastic bags. To maintain their stability and prevent premature degradation, they were kept in a dark, dry environment, preferably in a low-humidity area. To evaluate the effect of storage on sensor performance, ECL intensity was measured at four time points: immediately after preparation, after 1 day, after 1 week, and after 10 days. The results, shown in Figure S9 in the Supporting Information, indicate a progressive decrease of the signal: approximately 10% after 1 week and 18% after 10 days.

A 10% reduction after 1 week can be considered acceptable for short-term use, suggesting moderate degradation likely due to minor evaporation or reagent desorption. The 18% decrease after 10 days indicates a more substantial decline, which may affect sensitivity in applications requiring high precision. Nevertheless, the sensors remained functional, confirming that the system holds promise for short-term applications. However, these results also highlight the need for improved stabilization strategies or optimized storage conditions to ensure longer shelf life and consistent analytical performance.

Beyond evaluating storage stability, an important aspect of sensor performance, whose test is in progress, is its reproducibility. In fact, it reflects how consistently the sensor behaves when fabricated and used under the same protocol on different occasions.

## Conclusions

The present study demonstrates that office-paper based ECL sensors provide an optimal balance between sensitivity, reproducibility and ease of use. By leveraging the unique advantages of paper-based substrates in ECL sensor design, a ready-to-use analytical platform that is robust, low-cost, and suitable for storage was developed. By prestoring reagents directly on the sensor surface, the platform simplifies operation for the end user and enables portable, user-friendly devices, while office paper provides comparable performance to polyester flexible SPEs, maintaining signal stability and offering a robust, low-cost alternative for ECL sensor fabrication. This technical note aims to serve as a guide for readers in selecting appropriate substrates according to their specific ECL applications. Considering that enzymes and other biomolecules can be stably stored on paper, these substrates also offer the potential to preserve functional biomolecules, extending their applicability to ECL-based detection of biological targets.^13,33^

A key finding of this study is that paper-based ECL sensors can retain storage stability while remaining ready-to-use, highlighting the practical advantage of prestoring reagents on the sensor surface. The sustained ECL functionality of TPA drop-casted electrodes after short-term storage, indicates that these devices can serve as disposable, reliable platforms. This stability also suggests that paper substrates effectively preserve functional biomolecules, supporting the potential of these sensors for future applications in biomolecule detection, with opportunities to further enhance reproducibility and extend storage longevity.

Overall, this work advances portable, user-friendly, and eco-conscious ECL sensors. By integrating reagent immobilization, reproducible fabrication, and storage stability, it lays the groundwork for future developments in paper-based biosensing. These findings open pathways for applications in environmental monitoring, healthcare diagnostics, food safety, and POC testing, demonstrating the practicality and versatility of paper-based ECL platforms.

## Supplementary Material



## Notes

The Supporting Information includes a proposed mechanism of the paper-based ECL system (Figure S1), the electrochemical characterization of the SPEs employing the substrates described above (Figures S2–S3), the analytical characterization of the polyester-based platform for comparison (Figure S5), ECL intensities of the paper-based substrates (Figure S6, S8), sensitivity comparison for the three substrates employed (Figure S7), storage stability of the paper-based ECL platform (Figure S9) (PDF)
