# Supplementary material for: Exploring the Feasibility of Paper-Based Substrates for User-Friendly Electrochemiluminescent Sensors
Source: Anal Chem. 2026 Jan 30;98(5):3485–92. doi: 10.1021/acs.analchem.5c06606 (PMC12903066; doi:10.1021/acs.analchem.5c06606)
Supplement: Supplementary file 1 [file ac5c06606_si_002.pdf]

# SUPPLEMENTARY INFORMATION

## Exploring the feasibility of paper-based substrates for user-friendly electrochemiluminescent sensors

Panagiota M. Kalligosfyri[1,a,*], Luca Scognamiglio[1,a], Elena Sossich[2], Ningtao Cheng[3], Federico Polo[2,4,*], Stefano Cinti[1,5,6,*]

[1] Department of Pharmacy, University of Naples "Federico II", 80131 Naples, Italy
[2] Department of Molecular Sciences and Nanosystems, Ca' Foscari University of Venice, Via Torino 155, 30172 Venice, Italy
[3]School of Public Health, Zhejiang University School of Medicine, Hangzhou, Zhejiang 310058, China
[4] European Centre for Living Technology (ECLT), Ca' Bottacin, 30124, Venice, Italy
[5] Sbarro Institute for Cancer Research and Molecular Medicine, Center for Biotechnology, College of Science and Technology, Temple University, Philadelphia, PA 19122, USA
[6] Bioelectronics Task Force at University of Naples Federico II, Via Cinthia 21, Naples 80126, Italy
[a] These authors contributed equally.

*Email: panagiota.kalligosfyri@unina.it, federico.polo@unive.it, stefano.cinti@unina.it

**Table of content**

**Proposed mechanism of the paper-based electrochemiluminescent sensor** Page S1

**Figure S1. Proposed mechanism of the electrochemiluminescent sensor** Page S1

**Electrochemical characterization of the polyester- and the paper-based substrates** Page S1

**Figures S2-S4.** CVs of 5 mM $K_3[Fe(CN)_6]$. Page S1-S2

**Figure S5.** ECL calibration curve with the polyester-based SPEs. Page S3

**Figure S6.** ECL responses of the office and filter paper-based SPEs. Page S4

**Figure S7.** Comparison of the sensitivity for polyester- and paper-based SPE. Page S4

**Figure S8.** ECL responses of the office ready to use paper-based SPEs. Page S5

**Figure S9.** Stability test of the ECL output vs storage time for paper-based SPE. Page S5

## Proposed mechanism of the paper-based electrochemiluminescent sensor

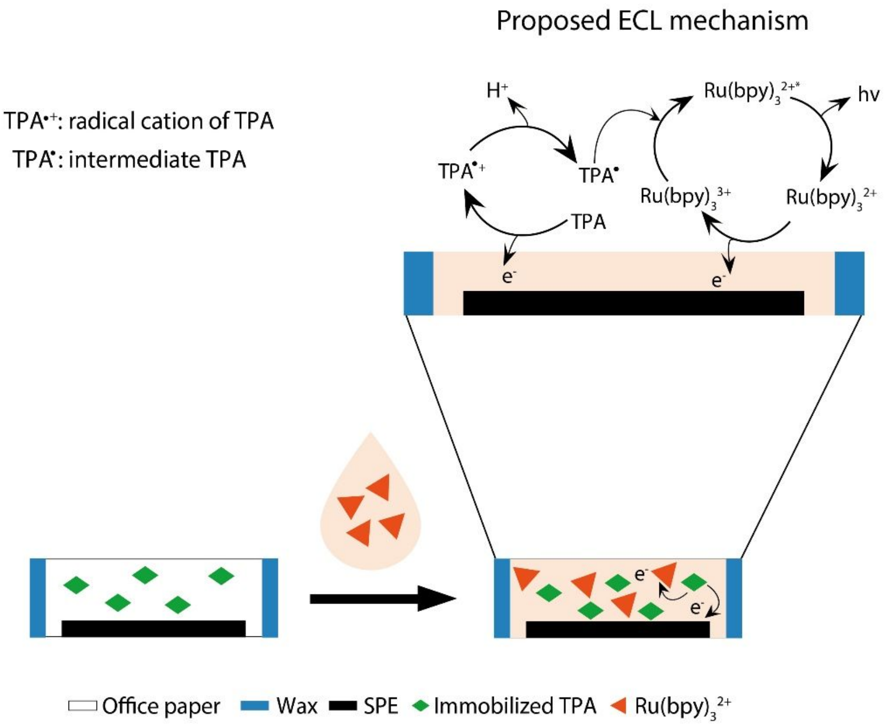


**Figure S1:** Schematic illustration of the electrochemiluminescence mechanism involving $Ru(bpy)_3^{2+}$ and immobilized tripropylamine (TPA) at the paper-based electrode surface, showing oxidation, excited-state formation, and light emission.

## Electrochemical characterization of the polyester and the paper-based substrates

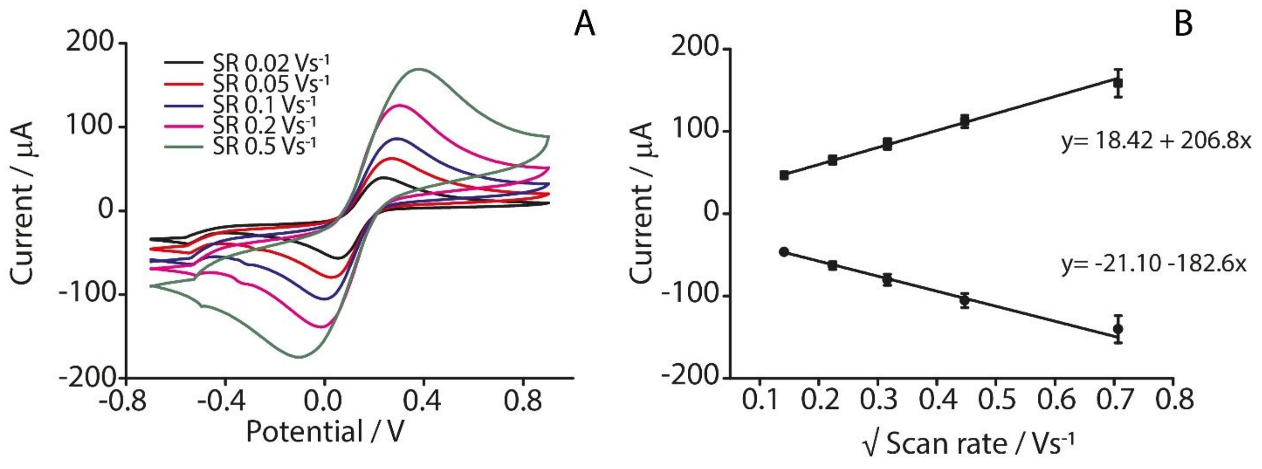


**Figure S2.** Electrochemical characterization of polyester-based SPE. A) CVs of 5 mM $K_3[Fe(CN)_6]$ in deionized water/100 mM KCl. Scan rates: 0.02, 0.05, 0.1, 0.2 and 0.5 V/s. B) Dependance of the anodic (solid black squares) and cathodic (solid black circles) current intensities on the square root of the tested scan rates. The experiments were performed in triplicates.

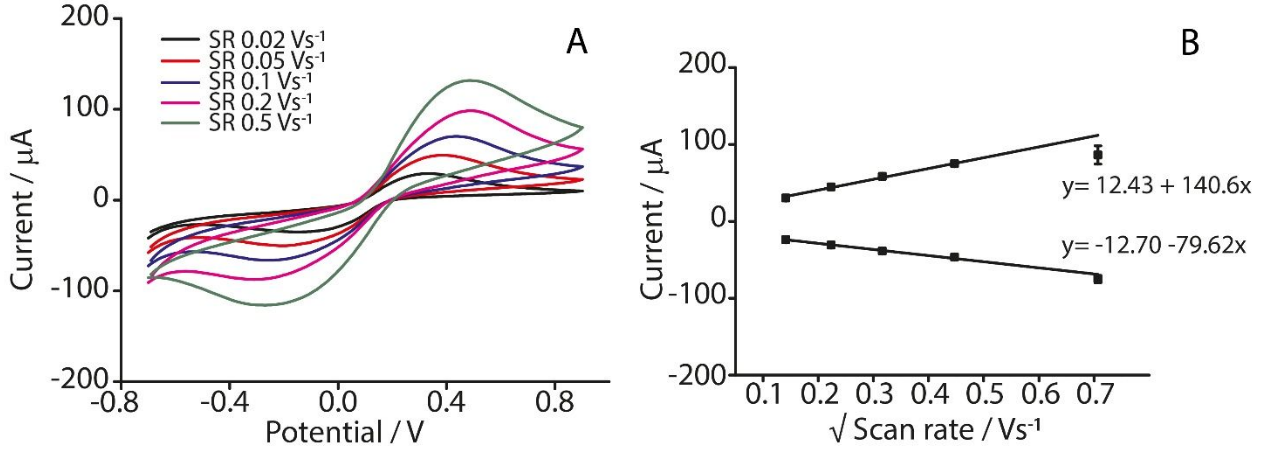


**Figure S3.** Electrochemical characterization of office paper-based SPE. A) CVs of 5 mM $K_3[Fe(CN)_6]$ in deionized water/100 mM KCl. Scan rates: 0.02, 0.05, 0.1, 0.2 and 0.5 V/s. B) Dependance of the anodic (solid black squares) and cathodic (solid black circles) current intensities on the square root of the tested scan rates. The experiments were performed in triplicates.

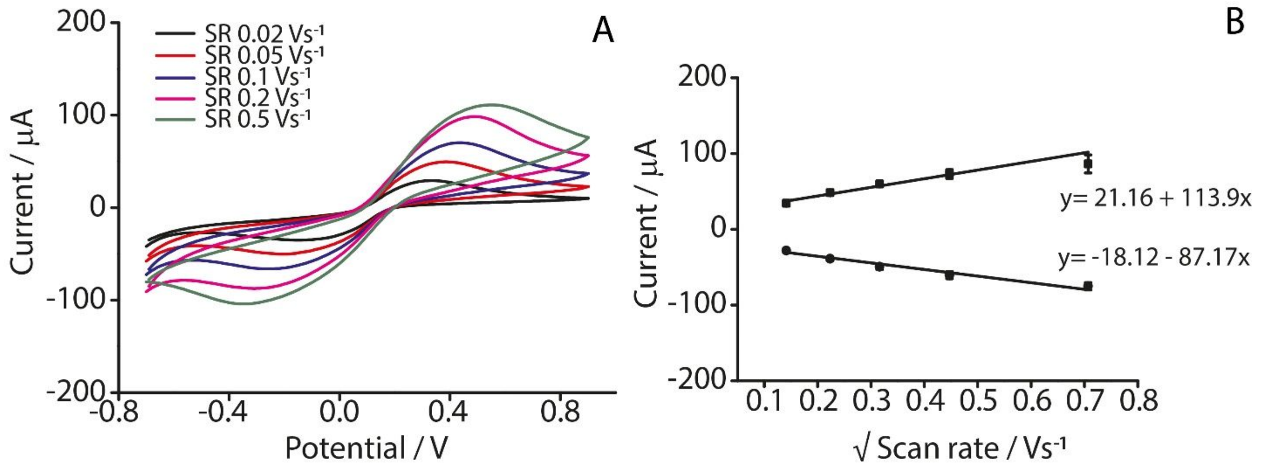


**Figure S4.** Electrochemical characterization of filter paper-based SPE. A) CVs of 5 mM $K_3[Fe(CN)_6]$ in deionized water/100 mM KCl. Scan rates: 0.02, 0.05, 0.1, 0.2 and 0.5 V/s. B) Dependance of the anodic (solid black squares) and cathodic (solid black circles) current intensities on the square root of the tested scan rates. The experiments were performed in triplicates.

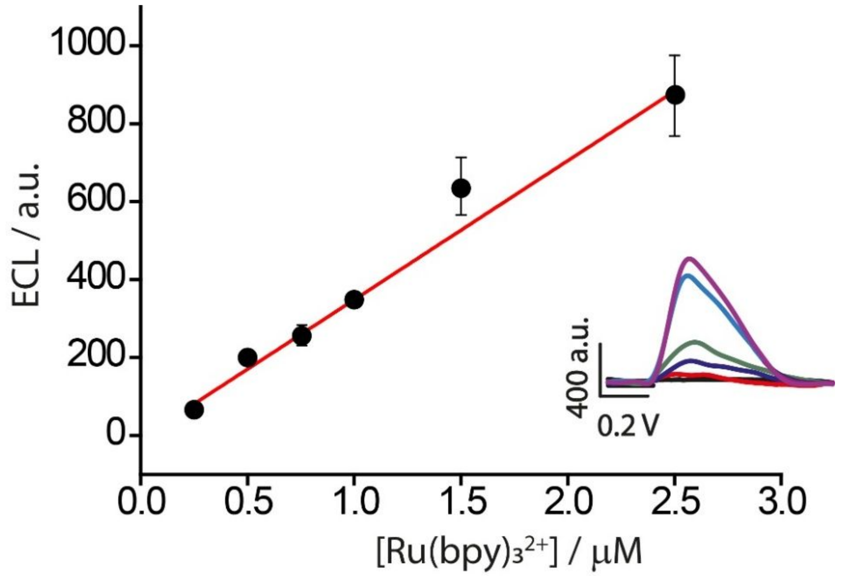


**Figure S5.** Calibration curve obtained for the polyester-based SPEs at increasing concentrations of $Ru(bpy)_3^{2+}$ in the range of 0.25-5 µM. Inset: ECL signal obtained with increasing concentrations

of $Ru(bpy)_3^{2+}$. The ECL measurements were performed in solution, in presence of 10 mM of TPA. The experiments were performed in triplicates.

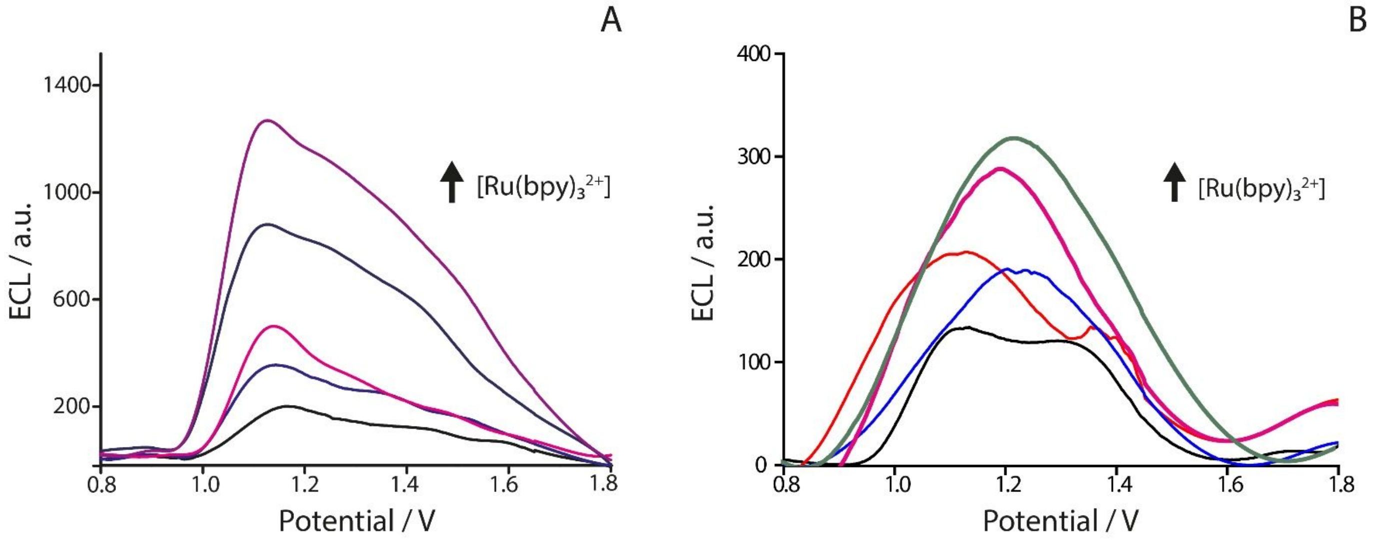


**Figure S6:** ECL response of (A) office paper and (B) filter paper obtained with increasing concentrations of $Ru(bpy)_3^{2+}$ in the range of 0.25–5 µM. The ECL signal increases with higher $Ru(bpy)_3^{2+}$ concentration, showing a distinct peak at 1.2 V.

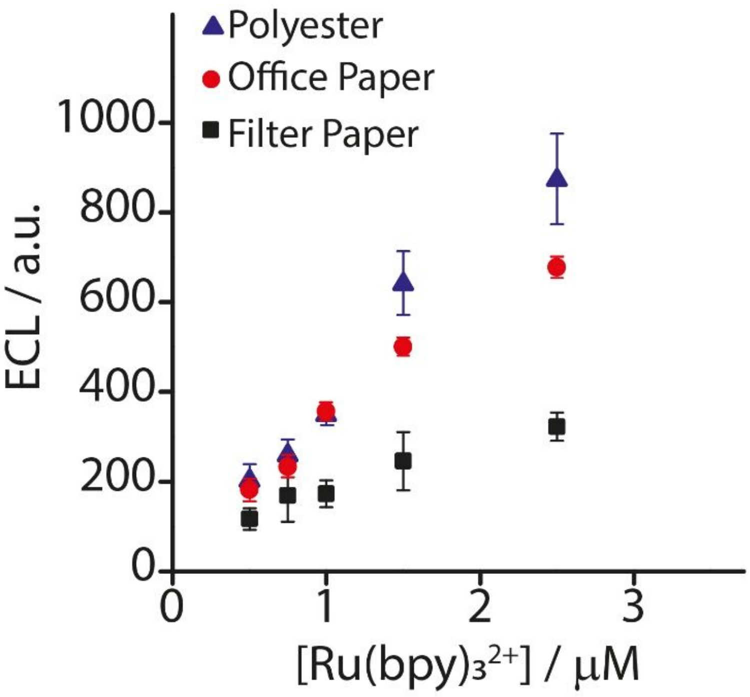


**Figure S7.** Comparison of the sensitivity (slope) of the three substrates: polyester, office paper and filter paper. The ECL measurements were performed in solution, in presence of 10 mM of TPA. The experiments were performed in triplicates.

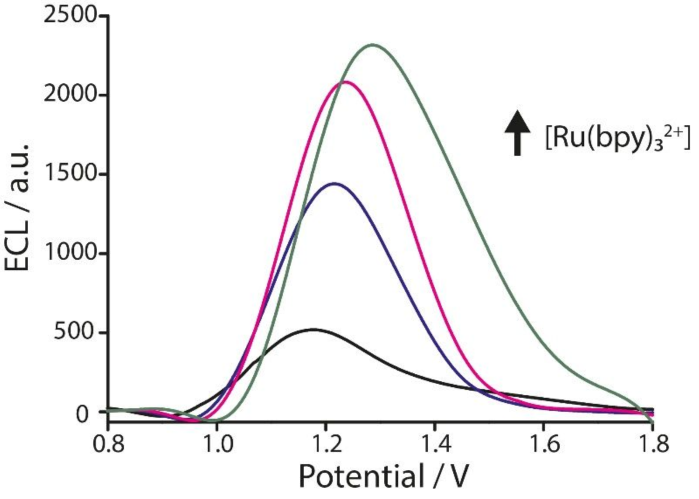


**Figure S8:** ECL response of office paper–based SPEs with 2 M immobilized TPA at increasing concentrations of $Ru(bpy)_3^{2+}$ in the range of 0.1–1 μM. The ECL signal increases with increasing $Ru(bpy)_3^{2+}$ concentration, exhibiting a distinct peak at 1.2 V.

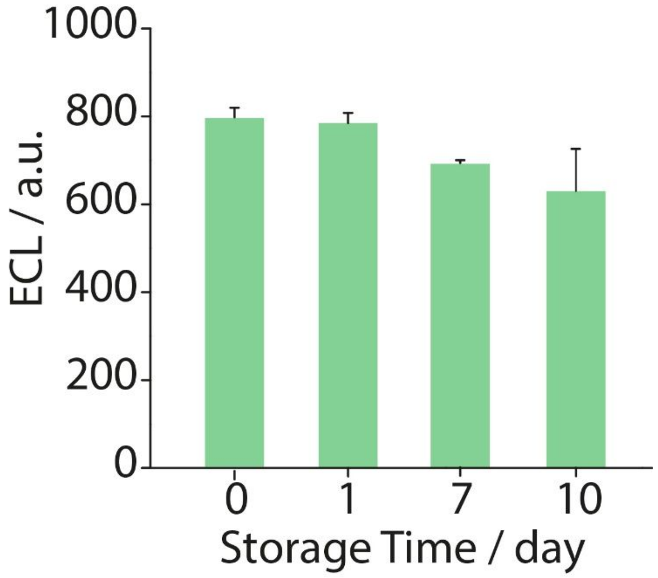


**Figure S9.** Ready-to-use paper-based platform's storage stability from day 0 to day 10 after storage. For this study 2 M of TPA were drop casted, towards a final concentration of 200 mM after reconstitution in presence 1 μM of ruthenium. The experiments were performed in triplicates.
